# Idiopathic Ventricular Arrhythmias Originating from Different Portions of the Coronary Venous System: Prevalence, Electrocardiographic Characteristics, Catheter Ablation, and Complications

**DOI:** 10.3390/jcdd9030078

**Published:** 2022-03-07

**Authors:** Yaoji Wang, Jiameng Shao, Bing Shen, Cheng Zheng, Jin Li, Que Xu, Yifan Lin, Jiafeng Lin

**Affiliations:** Department of Cardiology, The Second Affiliated Hospital and Yuying Children’s Hospital of Wenzhou Medical University, Wenzhou 325000, China; 719106@wzhealth.com (Y.W.); 721041@wzhealth.com (J.S.); 721036@wzhealth.com (B.S.); 211325@wzhealth.com (C.Z.); 205006@wzhealth.com (J.L.); 720128@wzhealth.com (Q.X.); 720125@wzhealth.com (Y.L.)

**Keywords:** radiofrequency catheter ablation, ventricular arrhythmias, cardiac electrophysiology, coronary venous system

## Abstract

(1) Background: To determine the prevalence, electrocardiographic characteristics, mapping, and ablation of IVAs arising from the CVS. (2) Methods: Detailed activation and pace mapping of the CVS IVAs was performed before attempted radiofrequency ablation (RFCA). (3) Results: The IVAs originating from the vicinity of the CVS represented approximately 5.27% (164/3113) of all IVAs; 94.51% (155/164) cases were accessed at the earliest identified site and 83.54% (137/164) IVAs were successfully ablated. The main coronary vein group had a relatively short procedure time, short fluoroscopy time, fewer radiofrequency lesions prior to success, and less Swartz sheath support. IVAs originating from the CVS had distinct ECG characteristics: Rs, RS or rS (with s or S) wave in lead V1 indicate the Vas arising from the proximal portion of the anterior interventricular vein (AIV) and summit-CV; Rs (with s or S) wave in leads V5–V6 indicate the Vas arising from the adjacent regions of the distal great cardiac vein 1 (DGCV_1_); positive wave (R, Rs or r) In lead I indicate the VAs ori”inat’ng from Summit-CV and posterior wall subgroup (including middle cardiac vein [MCV], posterior lateral vein [PLV], coronary sinus [CS]). Compared with the IVAs originating from the endocardial mitral annulus, a PdW > 45 ms, an IDT > 74 ms, and an MDI > 0.50 indicate a CVS origin of the IVAs. The common peri-procedure complications were CV dissection (6.45%, 10/155), CV rupture (1.29%, 2/155), coronary artery spasm (1.29%, 2/155), coronary artery stenosis (0.65%, 1/155), pericardial effusion (0.65%, 1/155) and tamponade (1.29%, 2/155). Stenosis of coronary arteries was not observed at the adjacent ablation site in the CVS during follow-up. (4) Conclusions: vAs arising from the CVS are not a rare phenomenon. Several ECG and procedure characteristics could help regionalize, map, and ablate the origin of IVAs from different portions of the CVS. RFCA within the CVS was relatively effective and safe.

## 1. Introduction

Idiopathic ventricular arrhythmias (IVAs), including premature ventricular complexes (PVCs) and ventricular tachycardias (VTs), are the most common arrhythmias observed in patients without structural heart disease. The incidence of a coronary vein system (CVS) origin ranged from 2.5% to 15% [1,2]. There are several reports of IVAs with the earliest activation and successful ablation inside the branches of the CVS [3,4]. The CVS provides a potential route for mapping and ablating vAs arising from the epicardial site. Few previous studies described the mapping and ablation of IVAs ablated in the transitional area from the great cardiac vein (GCV) to the anterior interventricular vein (AIV), however, without a clear distinction of ECG morphology, procedure characteristics of IVAs originating at different sites within the CVS.

The goal of the present study was to determine the prevalence, electrocardiographic characteristics, mapping, and ablation of idiopathic vAs arising from the CVS, meanwhile, the safety of this approach was also monitored.

## 2. Methods

### 2.1. Study Population

From October 2009 to March 2021, a total of 3113 patients (male 1288; age 49.43 ± 17.39 years, range 4–87 years old) presented for catheter ablation for IVAs at our hospital; 164 (5.27%) patients with IVAs in the vicinity of the CVS were enrolled. The site of origin was confirmed to be near the CVS when the mapping catheter positioned in the CVS showed the earliest activation time during arrhythmia with an excellent matching pace map (≥11/12 leads) obtained by pacing at the location. No enrollees had structural heart disease as assessed by routine biochemistry tests, X-ray, color echocardiography examination, exercise electrocardiogram testing, or cardiac catheterization with coronary angiography (CAG) or cardiac magnetic resonance when needed. Before the ablation, a 12-lead ECG was obtained, and 24-h ambulatory ECG monitoring (Holter) was carried out at least once. All antiarrhythmic drugs were withdrawn for at least five half-lives. The selection criteria of patients were the following: ① frequent or consecutive PVCs with mean count ≥ 10,000 times/24 h on Holter or with severe clinical symptoms; ② useless treatment with at least one antiarrhythmic drug; ③ no structural heart disease; ④ consent for the catheter ablation procedure. Each participant provided informed written consent. The Ethics Committee of the Second Affiliated Hospital of Wenzhou Medical College approved the study.

### 2.2. Electrophysiological Study and Radiofrequency Ablation

**Figure 1:**
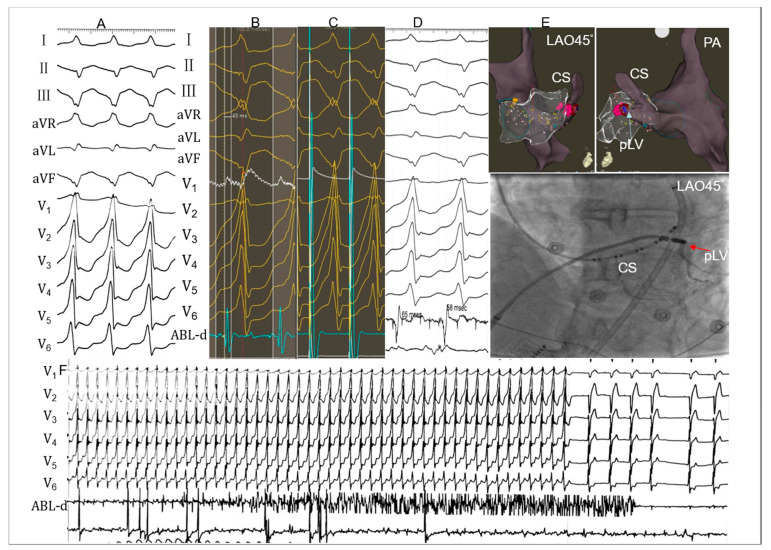
Example of a successful ablation of ventricular tachycardia (VT) originating from the posterior lateral vein (PLV). (**A**) Twelve-lead ECG morphology of the clinical VT. R wave in lead I, QS wave on leads II, III and aVF, r wave in lead V1, Rs wave on leads V5 and V6. (**B**) The local ventricular activation time recorded at the posterior wall of the endocardial mitral annulus (endo-MA) preceded the onset of the QRS complex by 43 ms and pacing this site led to a 95.6% concordance with the QRS complex of VT. (**C**) Cessation of the target VT is not observed within the 30s after radiofrequency initiation (pink dots in (**E**)). (**D**) Another local ventricular activation time recorded at PLV (the posterior wall of epicardium mitral annulus) preceded the onset of the QRS complex by 58 ms. (**E**) The fluoroscopic position of the successful ablation site was in the PLV. (**F**) Ablation was repeated at 43 °C, 30 W with a 60 mL/min irrigation rate. Cessation of the target VT was observed 15 s after radiofrequency initiation (red dots in Figure 1E). LAO = left anterior oblique; PA: postero-anterior. ABL-d, intracardiac bipolar electrograms; PLV, posterior lateral vein; CS, coronary sinus.

Electrophysiological studies and ablation were performed after discontinuation of all antiarrhythmic drugs for at least five half-lives. Figure 1 shows the procedure process. A 6-F decapolar catheter (4-mm interelectrode spacing; Inquiry LumaCath Fixed Diagnostic Catheter; St. Jude Medical, Saint Paul, MN, USA) was inserted from the right internal jugular vein and placed in the coronary sinus as distal as possible, which helped to thoroughly map the CVS. If clinical arrhythmias failed to occur spontaneously, intravenous isoproterenol (2–5 mg/min) was infused. An irrigated-tip ablation catheter was advanced to the right ventricle and coronary venous system via the antegrade transvenous approach and the left ventricle via the retrograde aortic approach. Activation mapping (CARTO; Biosense Webster, Diamond Bar, CA, USA) was preferentially used to identify the origin. Pace mapping was also performed to capture the ventricular myocardium at the site of earliest activation. A suitable target for ablation was selected based on the earliest activation times during the arrhythmia combined with pace mapping.

The right ventricular outflow tract (RVOT), LV endocardium, aortic cusp and CVS were mapped with the ablation catheter (or 6-F decapolar catheter) to identify the earliest site of ventricular activation during IVAs. All included patients whose earliest ventricular activation was observed in the CVS through systematic mapping with or without the best pace mapping score and the targeted IVAs disappeared during the ablation. When the ablation from the CVS failed, we ablated from the corresponding position of the endocardium, if IVAs disappeared post adjacent site ablation, an intramural origin of IVAs was considered due to a combination of endocardial and epicardial ablation which contributed to the final success. We excluded these cases from the study. If IVAs could not be abolished by adjacent site ablation, we still considered the CVS origin of IVAs according to the earliest site of ventricular activation.

The ideal target site of radiofrequency catheter ablation (RFCA) was determined using acceptable pace mapping (≥11/12-lead or 95% concordance of major and minor deflections) with the earliest local activation time. Once the origin of IVAs was found within the CVS. RFCA would be attempted using the irrigated-tip catheter in a power-controlled mode with a maximum temperature of 43 °C, preset power of 25–35 W, a flow rate of 30–60 mL/min, and impedance of 300 Ω. CAG was performed in all cases to investigate distance from the catheter tip to adjacent coronary arteries before RFCA through multiple postures during systole. Energy delivery was forbidden when the distance was less than 5 mm. Coronary blood supply was routinely evaluated before and after ablation. If IVAs were terminated or more PVCs and/or non-sustained ventricular tachycardia occurred during the initial 15 s ablation at the target site, radiofrequency delivery would continue for 60 to 180 s. Otherwise, other targets were sought. Figure 1 shows an example of the successful ablation of CVS IVAs.

We defined acute success as both the absence of spontaneous or provoked clinical VAs at the end of the procedure (by intravenous administration of isoproterenol and programmed stimulation) and the latter 48-h period post-ablation on Holter without antiarrhythmic drugs.

### 2.3. Anatomy of the CVS

A systematic understanding of IVAs originating from the CVS requires a thorough understanding of CVS anatomy. The use of coronary venography by retrograde injection of contrast medium into the coronary sinus usually allows a clear demonstration of the architecture of this system. The coronary venous tree presents a wide range of variation; it comprises the coronary sinus and great, middle, and small cardiac veins [5].

The coronary sinus (CS), which is located at the cardiac crux, originates in the right atrium and runs along the posterior and lateral parts of the mitral annulus. After crossing the posterior interventricular sulcus, it directly continues as the great cardiac vein (GCV) that runs around the anterior and lateral portions of the mitral annulus, GCV was divided into three segments (proximal, middle, and distal) by lateral vein (LV) and posterior-lateral vein (PLV), the distal great cardiac vein (DGCV) was divided into two regions: DGCV_1_ (epicardium of the anterior-lateral wall of the mitral annulus) and DGCV_2_ (proximal to the origin of the anterior interventricular vein [AIV]). The distal region of the DGCV continues as the AIV, which runs in the anterior interventricular sulcus and is parallel to the left anterior descending coronary artery (LAD). In some patients, coronary venography also shows a distinct branch between the aortic and pulmonary annulus, which is the extended tributary of the DGCV located distal to the origin of the AIV, known as the communicating vein of the left ventricular Summit (Summit-CV). The middle cardiac vein (MCV), also known as the inferior interventricular vein, starts at the apex of the heart adjacent to the posterior descending artery, runs in the inferior interventricular sulcus, and eventually empties into the coronary sinus.

**Figure 2:**
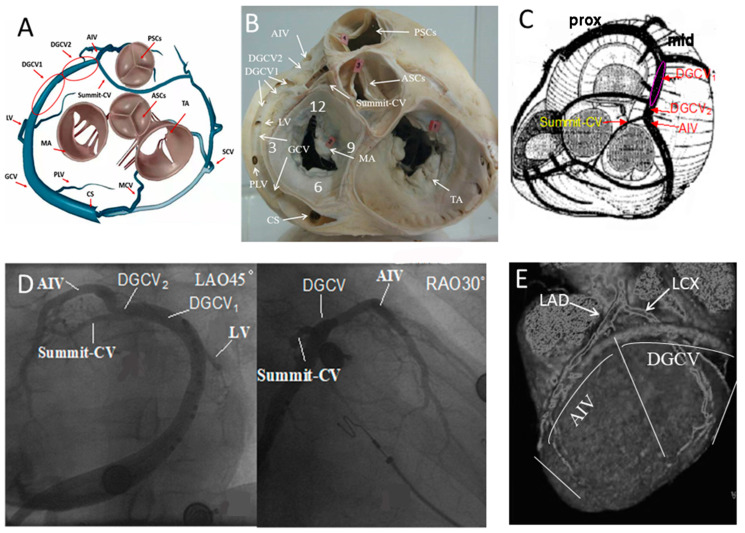
The architecture of the coronary venous system (CVS). (**A**,**C**), schematic view of the CVS; (**B**), posterior-anterior view of the endocardial and epicardial mitral annulus; (**D**), coronary venography allows clear visualization of the coronary venous architecture; (**E**), CT angiography of the anterior wall of the heart shows the anatomical relationship to the left anterior descending coronary artery (LAD) and the left circumflex coronary artery (LCX). Abbreviation: CS, coronary sinus; GCV, great cardiac vein; DGCV_1_, the distal great cardiac vein local in epicardium of the anterior lateral wall of the mitral annulus; DGCV_2_, the distal great cardiac vein proximal to the origin of AIV. MCV, middle cardiac vein; SCV, small cardiac vein; AIV, anterior interventricular vein; Summit-CV, the communicating vein in left ventricular Summit; PLV, Posterior lateral vein; LV, lateral vein; MA, mitral annulus; TA, tricuspid annulus; PSCs; pulmonary sinus cusp; ASC; aortic sinus cusp. RAO = right anterior oblique; LAO = left anterior oblique.

These veins, constituting the CVS, practically drain approximately three-quarters of coronary blood flow. The remaining venous return, which usually empties into the coronary sinus through the small cardiac vein, occasionally may infuse blood flow directly into the right atrium. The architecture of the CVS is shown in Figure 2. The anatomic structure of the CVS provides unique access to the left ventricular epicardium with minimal injury.

### 2.4. ECG Analysis

The following ECG features were assessed in patients with IVAs originating in various positions of the CVS, as previously described: (1) the QRS morphology of the VAs in all 12 leads; (2) the site of R wave transition in the precordial leads; (3) the axis deviation; (4) the QRS complex duration; (5) the pseudo delta wave (PdW, the interval from the beginning of the QRS complex to the earliest fast deflection in any precordial lead) time; (6) the intrinsicoid deflection time (IDT, the interval from the beginning of the QRS complex to the peak of the R wave in V2); and (7) the maximum deflection index (MDI, IDT divided by the QRS duration). ECGs were read by two expert cardiac electrophysiologists blinded to the site of origin; in case of discordance, a third electrophysiologist was consulted, and the ECG features were adjudicated according to the majority vote. Capital letters (Q, R, S) refer to relatively high-amplitude waves (>5 mm). Lowercase letters (q, r, s) refer to relatively low-amplitude waves (≤5 mm).

### 2.5. Follow-Up

Patients were followed up through regular clinic visits and 24-h Holter monitoring for the first month after RFCA. Computed tomography of the coronary arteries or CAG was performed at least once post-ablation. Follow-up echocardiography and Holter monitoring were performed 3 to 12 months post-ablation. ECG and Holter monitoring were performed whenever the patient had symptoms suggestive of recurrence of VAs.

### 2.6. Statistical Analysis

Categorical variables were expressed as case numbers and percentages. Measurement data were expressed as mean ± standard deviation. The Shapiro–Wilk test was used for normal distribution. The continuous variables were compared with a Student’s *t*-test for two groups and Analysis of variance (ANOVA) for >2 groups in case of normal distribution. Categorical variables were presented as numbers and percentages and were compared using the chi-square testor the Fisher’s exact test when appropriate. Receiver-operating characteristic curves were used to obtain the best values of sensitivity and specificity. Statistical analysis was performed using the SPSS Statistics software, version 20.0 (IBM, Armonk, NY, USA). Two-sided *p*-values < 0.05 were considered to indicate statistical significance.

## 3. Results

### 3.1. Clinical Characteristics

Between October 2009 and December 2020, 3113 patients with IVAs underwent RFCA at our center; 5.27% (164/3113; 95 men; mean age 55.46 ± 13.73 years) were found to have IVAs arising from different portions of the CVS; 82.93% (136/164) patients with epicardial arrhythmias had PVCs, and 14.02% (23/164) patients had non-sustained ventricular tachycardia (NSVT). The remaining patients had sustained ventricular tachycardia (SVT). The mean left ventricular ejection fraction (LVEF) was 67.90 ± 7.70%, and the left ventricular end-diastolic internal diameter (LVEDd) was 49.89 ± 6.17 mm. Twenty (12.20%) patients had slightly and moderately dilated left ventricles (LVEDd range 55–66 mm). According to the origin of the effective target (or target site with the earliest local activation time) in the CV, the 164 patients were divided into four groups: ① AIV group; ② Summit-CV group; ③ main coronary vein group (including DGCV_1_, DGCV_2_, GCV, and CS); and ④ adjacent branches of the CVS group (including LV, PLV, and MCV). The clinical characteristics of the patients are shown in Table 1. There were no significant differences in clinical characteristics, including age, sex, history, type of arrhythmia, VA burden, LVEF, or LVEDd.

### 3.2. Mapping and Ablation

The mapping and ablation outcomes are shown in Table 2. The IVAs were acutely abolished in 137 of 164 (83.54%) patients by RFCA within the CVS. Successful ablation ratio was 72.97% (27/37) for AIV IVAs, 73.68% (14/19) for Summit-CV IVAs, 89.55% (60/67) for DGCV_2_ IVAs, 92.85% (26/28) for DGCV_1_ IVAs, 50.00% (1/2) for the GCV IVAs, 100.00% (2/2) for LV IVAs, 100% (1/1) for CS IVAs, 80.00% (4/5) for the MCV IVAs, and 66.67% (2/3) for PLV IVAs.

The rate of acute success in the main coronary vein group was higher than in the AIV groups (89.80% vs. 72.97%, *p* < 0.05). The ventricular activation in the CVS during targeted arrhythmias preceded the QRS onset by 34.91 ± 4.43 ms, and the mean of pace mapping concordance was 97.26 ± 2.83%, the average duration from onset of RF energy to the disappearance of IVAs was 7.52 ± 3.43 s. There was no significant difference in activation time (*p* > 0.05), pace mapping concordance (*p* > 0.05) and duration from onset of RF energy to the disappearance of IVAs among four groups; 94.5% (155/164) cases were accessed at the earliest identified site. The accessibility of the earliest identified site in the main coronary vein group was higher than those in the AIV groups (98.0% vs. 86.5%, *p* < 0.05). The ratios of Swartz sheath support were similar between AIV and Summit-CV groups (*p* > 0.05) but were relatively lower in the main coronary vein group (*p* < 0.05). During the mapping and ablation, procedure duration, fluoroscopy duration, and RF lesions prior to success in VAs of the main coronary vein were significantly shorter than in AIV, Summit-CV, and adjacent branches of the CV group (all *p* < 0.05); 84.8% (139/164) patients succeeded in pace mapping in the CVS, two patients had the atrium activated during pacing in the DGCV as the vein was close to the side of the atrium, 23 patients could not be captured the ventricle; 6.71% (11/164) cases increased the upper limit temperature setting to promote enough energy delivery due to high impedance, three cases in DGCV_2_, three cases in AIV and two cases in Summit-CV, three cases (two in DGCV_2_ and one in AIV) set the upper limit temperature to 45 °C and one case in DGCV_2_ set the upper limit temperature to 48 °C after increasing the upper limit temperature, the energy delivery could reach preset power (25–35 W), we give up ablation in the remaining seven cases because the energy delivery was only 10–12 W after setting the upper limit temperature to 48 °C.

In the 18 failed cases, failure was due to proximity to a major coronary artery in eight patients; high impedance prevents energy delivery in seven patients, one patient with diaphragmatic capture during high-output pacing in LV, and two patients with atrium activated during pacing in DGCV.

### 3.3. Angiography Results

The distance between the site of origin and the coronary artery was measured at the end of systole, which measured the distance between the head of the ablation catheter and the closest major coronary arteries through multiple postures. The site of origin was located a mean of 6.64 ± 1.17 mm from the closest major coronary artery, including 5.96 ± 0.86 mm in the AIV group, 6.31 ± 0.91 mm in the Summit-CV group, and 6.91 ± 1.11 mm in the main coronary vein group. The distance from the site of VAs origin to the coronary artery in the AIV group was relatively shorter than the main coronary vein group (*p* < 0.05).

### 3.4. ECG Characteristics

The 164 CVS IVAs patients were divided into two groups according to their location relative to the epicardial mitral annulus: ① 162 (98.78%) cases were around epicardial mitral annulus and ② only two (1.22%) cases were distal to the epicardial mitral annulus.

**Figure 3:**
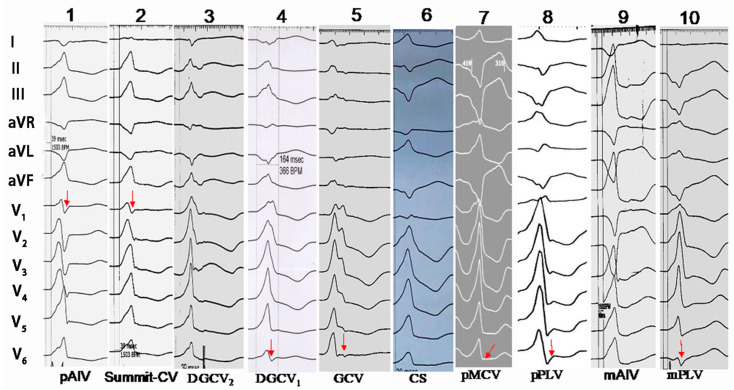
The QRS morphology of idiopathic ventricular arrhythmias (IVAs) originating from the CVS. pAIV, the proximal portion of the anterior interventricular vein; mAIV, the mid portion of the anterior interventricular vein; pMCV, the proximal portion of the middle cardiac vein; pPLV, the proximal portion of the posterior lateral vein; mPLV, the mid portion of the posterior lateral vein.

The characteristics of QRS morphology in different regions of CVS are shown in Table 3 and Figure 3. In the around epicardial mitral annulus group, the QRS morphology of VAs originating from the anterior septum (the proximal portion of AIV [pAIV] and Summit-CV) to anterior wall (DGCV_2_ and DGCV_1_) to lateral wall (GCV and the proximal portion of LV [pLV]) to the posterior wall (the proximal portion of MCV [pMCV], the proximal portion of PLV [pPLV], CS) of epicardium mitral annulus was as follows: ① in lead I, the QRS morphology demonstrated an rs→QS→rs→R wave. The Summit-CV and posterior wall subgroup VAs showed dominantly positive (R, Rs or r) waves (74.07%, 20/27); the dominantly negative wave was observed in the other regions: only 2.22% (3/135) demonstrated positive waves, and 97.78%(132/135) demonstrated negative waves (rS, rs, qr, qs or QS) (74.07% versus 2.22%, *p* < 0.001); ② in leads II, III and aVF, the R wave amplitude gradually decreased and S wave amplitude gradually increased. The QRS morphology progressed from R to RS (Rs) to QS wave. The reverse held for leads aVL and aVR, from QS to R wave; ③ in lead V1, pAIV and Summit-CV 92.73% (51/55) demonstrated Rs, RS or rS (with s or S) waves; in the other regions S or s wave was seldomly seen, only 8.41% (9/107) cases with s or S wave, 91.59% (98/107) demonstrated R (without S or s) wave (92.73% versus 8.41%, *p* < 0.001); ④ in leads V5~V6, in the DGCV_1_, lateral wall, and posterior wall subgroup 77.50% (31/40) demonstrated Rs (with s or S) waves; in DGCV_2_, Summit-CV, and pAIV, only 7.38% (9/122) demonstrated Rs (with s or S) waves, 92.62% (113/122) demonstrated monophasic R (without S or s) waves (77.5% vs. 7.38%, *p* < 0.001). There were only two cases in the distal mitral annulus group. One case arose from the mid portion of the AIV (mAIV), the morphology of the QRS was similar to pAIV IVAs and a demonstrated deeper QS (without r) wave in leads V1 and V2. The other case arose from the mid portion of the PLV (mPLV), it was the rs wave in lead I, the QS wave on leads II, III and aVF, R wave in leads aVL/aVR, R wave in lead V1, Rs wave on lead V5, and rS wave in lead V6.

We performed ECG identification of the IVAs originating from the CVS based on these results. The morphology of Rs, RS, or rS (with S/s wave) in lead V1 had a sensitivity of 92.73%, a specificity of 91.59%, a positive predictive value (PPV) of 85.00%, and a negative predictive value (NPV) of 96.08% to predict the VAs originating from the pAIV and Summit-CV. The morphology of Rs (with S/s wave) in leads V5–V6 indicated the VAs originating from the DGCV_1_, lateral wall and posterior wall subgroup, with sensitivity, specificity, PPV, and NPV were 77.50%, 92.62%, 77.50%, and 92.62%, respectively. The morphology of the positive wave (R, Rs or r) in lead I indicates that in the VAs originating from Summit-CV and the posterior wall subgroup, the sensitivity, specificity, PPV, and NPV were 74.07%, 97.78%, 86.96%, and 94.96%, respectively (Table 4).

### 3.5. Comparison ECG Characteristics of IVAs Originating from the CVS and the Endocardial Mitral Annulus (Endo-MA)

**Figure 4:**
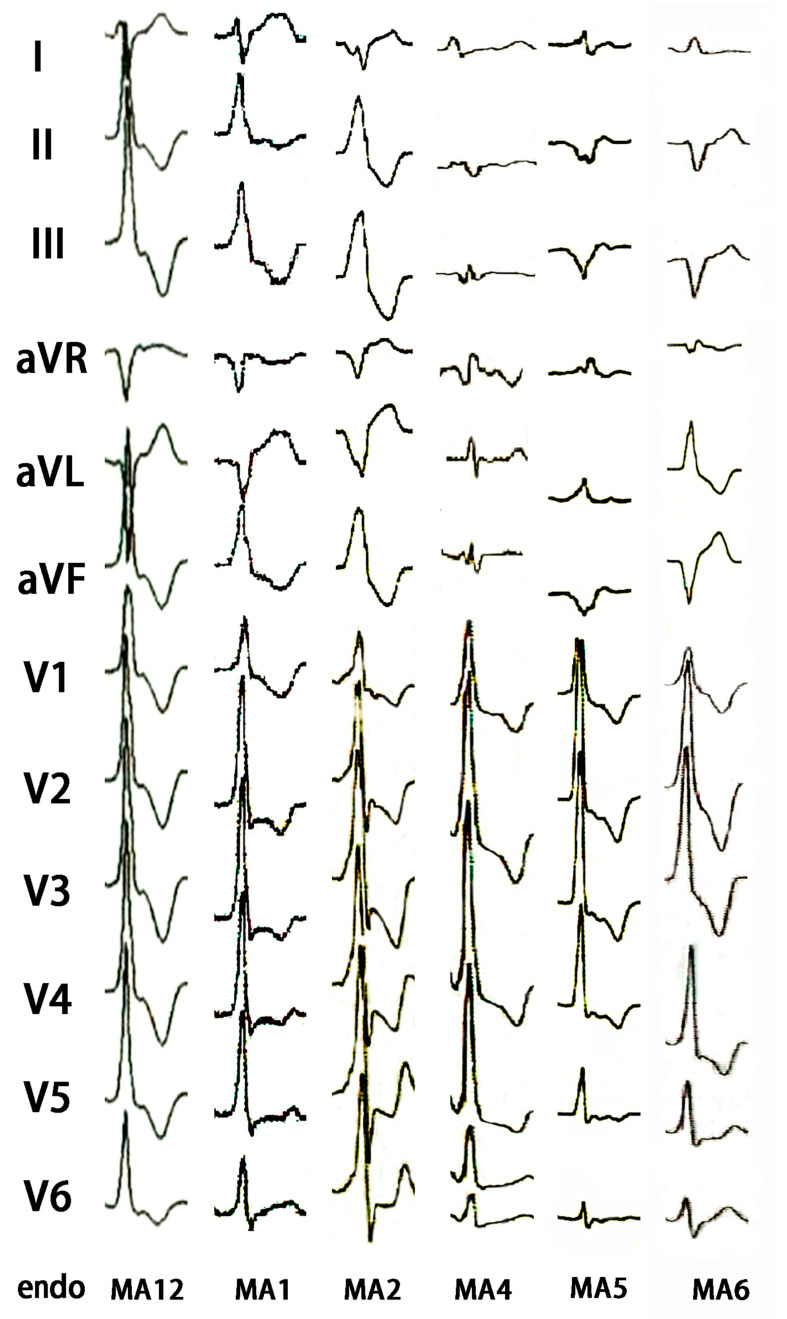
The QRS morphology of IVAs originating from the endocardial mitral annulus(endo-MA).

The patients with IVAs from the CVS were compared with 41 endo-MA IVAs patients originating from 12 o’clock to 3 o’clock to 6 o’clock (endo-MA12 to endo-MA3 to endo-MA6). The characteristics of the QRS morphology of the IVAs originating from the epicardial mitral annulus were almost identical to those of the IVAs arising from the adjacent endo-MA (Figure 3 and Figure 4).

**Figure 5:**
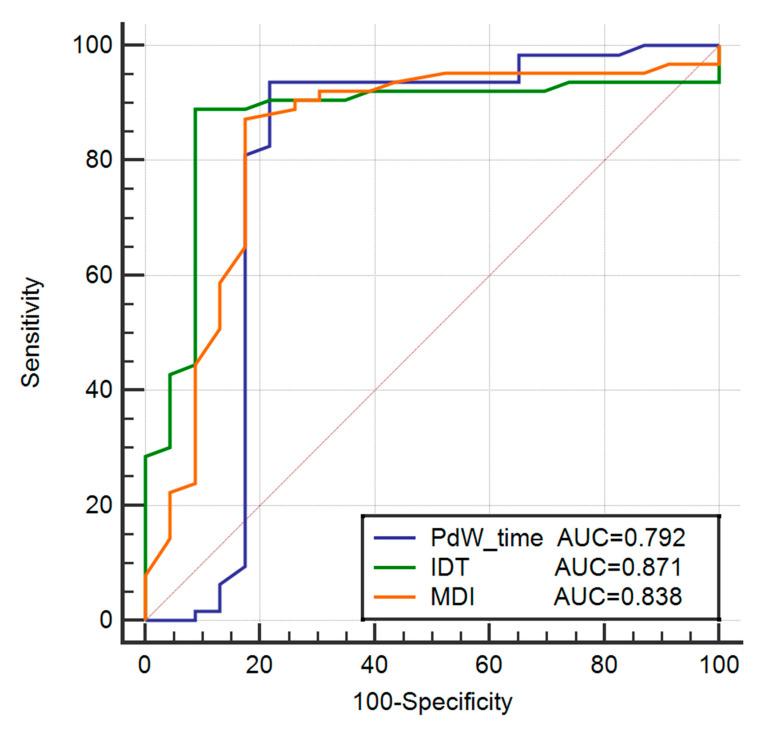
The ROC curve for diagnosing IVAs originating from the CVS. Pdw, pseudo delta wave; IDT, intrinsicoid deflection time; MDI, the maximum deflection index; AUC, Area Under Curve.

As shown in Table 5, the PdW time (61.05 ± 9.88 ms), IDT (90.14 ± 19.79 ms) and MDI (0.56 ± 0.09) were significantly longer in patients with IVAs arising from the CVS compared with the adjacent endo-MA, PdW time (49.41 ± 18.38 ms), IDT (65.76 ± 13.07 ms) and MDI (0.47 ± 0.09). No significant differences in QRS duration were found when the IVAs arising from the CVS were compared with the adjacent endo-MA. Receiver-operating characteristic (ROC) analyses determined the best cutoff value of the PdW time, IDT, and MDI that distinguished the CVS group from the adjacent endo-MA group was >45 ms, >74 ms, and >0.50, the sensitivity, specificity, PPV, and NPV of the PdW time, IDT, and MDI were 93.6%, 78.2%, 92.2%, 81.8%; 88.9%, 91.3%, 93.33%, 75.0% and 87.3%, 82.6%, 87.3%, 82.6%, respectively (Figure 5).

### 3.6. Procedure Complications

**Figure 6:**
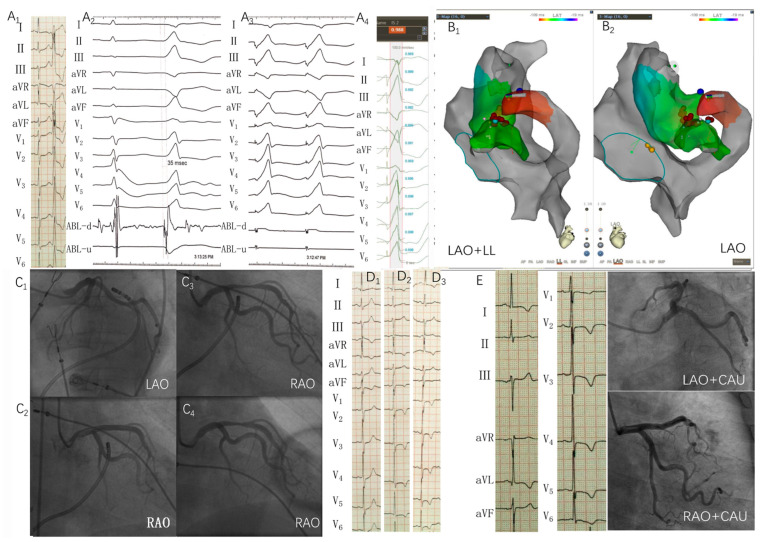
Example of ablation of IVAs originating from the Summit-CV. Epicardial mapping and ablation were performed in a 66 year-old woman with clinical IVAs. No structural heart disease was evident on echocardiography. Preoperative surface ECG ((**A_1_**), activation mapping (**A_2_**) and pace mapping (**A_3_**) demonstrated rS wave on lead I, tall R wave on leads II, III, and aVF, and R wave in lead V_1_–V_6_. The local ventricular activation time recorded at Summit-CV preceded the onset of the QRS complex by 35 ms (**A_2_**) and pacing this site led to a 98.8% concordance with the QRS complex of IVA (**A_4_**). Activation mapping (**A_2_**) shows the intracavity bipolar electrogram (ABL-d) recorded from systolic high-amplitude potential to presystolic high-amplitude potential once mapping at ideal target site. (**B_1_**,**B_2_**) Electroanatomical map of the aortic sinus of Valsalva, the summit of the left ventricle, and Summit-CV. (**C_1_**,**C_2_**) show the fluoroscopic position of the ablation catheter site and coronary angiography (CAG). The middle segment of the left anterior descent branch (LAD) is located about 5.16 mm from the proximal edge of the ablation electrode at the ideal ablation site (note that the distal electrode of the ablation catheter is 2.67 mm [8-F] in diameter). Ablation was repeated with 43 °C, 25 W with an irrigation rate of 30 mL/min. The patient experienced temporary chest pain at 13s during ablation; immediate coronary angiography showed around 50% stenosis of the middle branch of the LAD (**C_3_**). Intracoronary nitroglycerin was given with incomplete response (**C_4_**), considering distal flow remained good, a stent was not inserted. Aspirin, clopidogrel, and isosorbide mononitrate were given after the procedure. (**D_1_**–**D_3_**) demonstrates changes in ECG in the following days. T wave voltage showed widespread decreases, and inverted T waves formed in lead I, aVL, and V_1_–V_6_. After 22 days, the patient experienced pericardial tamponade and was managed successfully by percutaneous pericardiocentesis and glucocorticosteroid treatment without further fluid reaccumulation. The patient remained asymptomatic from the coronary injury during 48 months of follow-up. Three years later, the patient presented with chest pain. Surprisingly, ECG still revealed inverted T waves in leads I, aVL, and V_1_–V_6_ I; however, coronary angiography showed the middle segment of the LAD was patent (**E**).

Fifteen patients suffered from peri-procedure complications (Table 6). In three cases, acute coronary injury presenting with ST-segment elevation immediately after ablation was recognized during the procedure. One case involved the LAD, one case involved the first diagonal branch (D1), and another involved the LCX. The mean distance from the target to the coronary artery was 5.10 ± 0.06 mm. Two cases were diagnosed with coronary spasms and succeed to respond to nitroglycerin (ST-segment was elevated nearly back to normal the following day). Another case in the LAD was managed unsuccessfully using nitroglycerin, the details are shown in Figure 6. Two patients suffered from coronary vein rupture, which induced pericardial tamponade in one patient and pericardial effusion in one patient, managed successfully by pericardiocentesis. Coronary vein dissection occurred in ten patients.

### 3.7. Follow-Up

Patients were followed up for 31 ± 27 months (range 3–72 months). All patients finished 24-h Holter monitoring for the first month, there was a more than 80% reduction of the PVCs burden demonstrated by 24-h Holter (24476 ± 13429 vs. 354 ± 226; *p* < 0.001) and 11 of 164 patients were lost of follow-up during 3 to 12 months post ablation. Three patients had recurrent ventricular arrhythmia. Redo procedures were successful in one patient and two patients remained on antiarrhythmic medications. Coronary arteries were evaluated using CT angiography or invasive CAG in 54 patients: ① no coronary stenosis was found in 18 patients; ② 34 patients showed coronary atherosclerosis. ③ Two patients showed severe coronary arteries stenosis. One patient had 75% stenosis in the distal segment of LAD, and the other patient had 60% in the distal LCX; however, the stenosis site was remote from the ablation site in the CVS.

## 4. Discussion

### 4.1. Main Findings

We described a group of patients with IVAs originating from the vicinity of the CVS in the absence of structural heart disease. We focused on prevalence, distinctive ECG morphology of different origin sites, catheter ablation through the CVS, and complications. The CVS IVAs were characterized as follows:

1. The IVAs originating from the vicinity of the CVS represented approximately 5.27% of all IVAs.
2. Rs, RS, or rS (with S/s wave) in lead V1 indicate the VAs originating from the pAIV and Summit-CV. Rs (with S/s wave) in lead V5–V6 indicate the VAs originating from the proximal regions of DGCV_1_ (including DGCV_1_, lateral wall, and posterior wall subgroup). Positive wave (R, Rs or r) in lead I indicate the VAs originating from Summit-CV and posterior wall subgroup. Compared with the IVAs originating from the endocardial mitral annulus, we also found that a PdW > 45 ms, an IDT > 74 ms, and an MDI > 0.50 indicate a CVS origin of IVAs.
3. The main coronary vein group has a relatively short procedure time, short fluoroscopy time, fewer RF lesions prior to success, and less Swartz sheath support. Although the rate of acute success in the main coronary vein group and LV, PLV, MCV group was tended to higher than Summit-CV and AIV group but not statistically significant. Additionally, sites in the AIV were more frequently in proximity to major coronary artery (5.96 ± 0.86 mm). The common limitation was the inability to access the earliest site of activation (as defined by the decapolar mapping catheter) with the ablation catheter (5.59%), proximity to coronary arteries (4.88%), inadequate power delivery (4.27%), pacing mapping activates atrium (1.22%) and phrenic nerve stimulation (0.61%).
4. The common peri-procedure complications were CV dissection (6.45%, 10/155), CV rupture (1.29%, 2/155), coronary artery spasm (1.29%, 2/155) coronary artery stenosis (0.65%, 1/155), pericardial effusion(0.65%, 1/155) and tamponade(1.29%, 2/155). Pericardial effusion and tamponade often occur after coronary artery and vein injury. The peri-procedure acute complications were often managed successfully by proper treatment. No evidence supported RFCA in the CVS resulted in damage to the coronary artery during long-term follow-up in 54 patients.

### 4.2. ECG Characteristics of IVas Originating from the CVS

Few previous studies described the ECG features of IVAs ablated in the transitional area from the GCV to the AIV [6,7], however, without a clear distinction of QRS morphology of IVAs originating at different sites within the CVS. Most CVS IVAs originated adjacent to the epicardial mitral annulus. ECG characteristics of IVAs originating from the different portions of the CVS were different and can help regionalize the origin of these arrhythmias.

Several previous studies have described different criteria for identifying an epicardial origin of IVAs. PdW, IDT, and MDI have emerged as highly sensitive and specific predictors of an epicardial origin [1,7,8]. Berruezo et al. [8] demonstrated that the epicardial origin of activation produces a wider PdW in patients with structural heart disease. Daniels et al. [1] reported that MDI ≥ 0.55 indicated an epicardial origin with a sensitivity of 100% and specificity of 98.5%. In the present study, we found that a PdW > 45 ms, an IDT > 74 ms, and an MDI > 0.50 indicate VAs originating from the epicardia. The differences between endocardially- and epicardially-stimulated QRS are probably due to fast depolarization of the ventricles along with the specialized conducting system when the stimulus is delivered in the epicardium; when initial activation occurs in the epicardium, the intramyocardial delay of conduction produces a wider pseudo delta wave and IDT and a greater MDI.

### 4.3. Ablation of the CVS IVAs

The outcome of the ablation procedure was associated with the location of the site of origin within the CVS. Comparing the AIV and Summit-CV groups, the main coronary vein group had relatively shorter procedure time, shorter fluoroscopy time, fewer RF lesions prior to success, and less Swartz sheath support. The most likely explanations for the observed discrepancy are as follows. ① An acute angle between AIV and DGCV_2_ would prevent the ablation catheter from reaching the proximal AIV. The Summit-CV is a distinct CV located between the aortic and pulmonary annulus. The very narrow lumen of this vessel usually limits the detailed mapping and ablation in this region. Above all are potential anatomic factors preventing catheter ablation of the AIV and Summit-CV. ② A previous study [2] showed that sites in the AIV were more frequently close to the left anterior descending coronary artery (79%), as the two vessels course together in the anterior interventricular groove. While proximity to coronary arteries was slightly less common in the GCV, it occurred in 40% of cases. In our practice, sites in the AIV were more frequently close to the LAD (5.96 ± 0.86 mm). Mapping and ablation of IVAs in the AIV could be more challenging. ③ We are more likely unable to deliver sufficient energy in the AIV or Summit-CV VAs, likely due to smaller vein caliber and higher impedance than the main coronary vein group.

Our center has the following measures to improve the success rate. ① Swartz sheath support approach ensured a powerful backup force for ablation catheter, which could assist catheter tip to overcome partial anatomic impediments of the coronary venous system and reach the target sites in the CVS more easily. Sometimes due to the blockage of the Vieussense valve, even with the support of Swartz sheath, the catheter tip could not be inserted into the middle part of GCV, which could be guided by a super smooth guidewire and Judkin’s catheter. The Swartz sheath was advanced alternately and passed the Vieussense valve, then the guidewire and Jukin’s catheter was exchanged with an ablation catheter to perform mapping and ablation in the distal GCV. ② The use of irrigated catheters at the coronary sinus was useful for delivering effective RF ablation power in the low-flow venous system to reduce the impedance. We summed up the following graded treatment strategies: a. Reset the upper limit impedance of the ablation instrument to 300 Ω. b. Accelerate the irrigation rate (maximum 60 mL/min); c. Turn off the upper limit impedance. d. Change the ablation instrument from Stockert EP shuttle (SES, Biosense Webster, CA, USA) to IBI-1500T11 (T11, St. Jude Medical, Saint Paul, MN, USA). e. Manually irrigate saline instead of pump injection. f. Increase the upper limit temperature of the ablation instrument to 45 °C (The maximum temperature we had used was 48 °C). Although no peri-procedure complications were found in our center, the safety needed to be further confirmed in the larger group. ③ The CS/GCV and LCX, AIV and D1, the MCV and the posterior branch of the left ventricle artery had a higher frequency of intersecting [9]; therefore, preoperative coronary angiography is necessary to clarify the relationship between the RF target and the coronary artery. Energy delivery was forbidden when the distance was less than 5 mm. During angiography, cardiac motion can make the distance and time spent between the target and coronary arteries difficult to assess; we suggest it is relatively safe to measure the distance during systole.

### 4.4. Complications of Ablation

Although previous case reports and series demonstrated that the transvenous approach is safe and effective [6,10,11], it is essential to note that complications caused by thermal or mechanical injury can occur, including rupture or dissection of coronary veins and spasm or stenosis of neighboring coronary arteries. For coronary artery injury with CVS ablation secondary to the close to the proximity coronary arteries, the mean of the distance from the target to the coronary artery in the acute coronary injury group was less than non-acute coronary injury (5.10 ± 0.06 mm vs. 6.64 ± 1.17 mm, *p* < 0.000).

Coronary veins injuries were often caused by improper catheter operation in the lumen. Peri-procedural acute complications often can be managed successfully by proper treatment. In a porcine model study on the chronic effects of RF energy delivery adjacent to coronary arteries, Viles Gonzalez et al. [12] reported that the absence of evident acute coronary injury does not preclude the development of significant artery stenosis after ablation. Our study did not find significant coronary stenosis adjacent to the ablation site in the CVS after 31 ± 27 months follow-up. Only one patient showed an ECG with inverted T waves in lead I, aVL, and V1–V6; however, CAG showed the coronary arteries were patent. One may hypothesize that this variation in T waves could be related to the damage of myocardial microcirculation. However, Pons et al. [13] reported a 24-year-old man with idiopathic VT who presented to the hospital complaining of exercise-induced chest pain 2 years after ablation, and coronary angiography showed severe chronic left main artery occlusion. Hence, more data are needed to confirm the long-term safety of RFCA in the CVS.

### 4.5. Limitations

First, this was a single-center study; although the sample size was relatively large, there may still be sampling errors. We expect that large multi-center cohort studies will be conducted in the future to validate our findings. Second, the anatomic structures in the heart are continuous and not discrete, while CVS categorization is relatively subjective. Thus, any arrhythmia originating in the border between categories can adversely affect the ECG-based algorithm. Third, only 32.93% (54/164) of people were followed up by CAG or CTA, long-term safety in coronary artery needs to be verified further and we are positioning to conduct relevant clinical research for evidence-based best results.

## 5. Conclusions

IVAs with the earliest activation in the CVS comprise 5.78% of all IVAs. Electrocardiographic and procedure characteristics of IVAs originating from different portions of the CVS differ, and this could assist in localizing, mapping, and ablating IVAs originating from the CVS. RFCA within the CVS was relatively effective and safe. We did not observe long-term side effects in coronary arteries ablated via the CVS.

## Figures and Tables

**Table 1:** Comparison in general clinical situation among different portions of the CVS.

Baseline Characteristics	Overall (n = 164)	AIV (n = 37)	Summit-CV (n = 19)	DGCV_1_, DGCV_2_, GCV, CS (n = 98)	LV, PLV, MCV (n = 10)	*χ*^2^/*F*	*p*
Age, years old	55.46 ± 13.73	52.95 ± 11.45	54.88 ± 14.56	55.96 ± 15.94	55.24 ± 14.22	1.122	0.342
Gender (men), n%	57.93% (95/164)	62.16% (23/37)	57.89% (11/19)	57.14% (56/98)	50% (5/10)	0.643	0.901
Duration of symptoms, years	2.87 ± 1.85	2.73 ± 2.01	3.39 ± 1.93	2.69 ± 1.91	1.89 ± 1.79	1.533	0.208
BMI, kg/m^2^	25.24 ± 2.92	25.56 ± 2.33	25.95 ± 4.69	25.06 ± 2.71	24.56 ± 2.68	0.698	0.561
Smoking, n (%)	17.68% (29/164)	18.92% (7/37)	21.05% (4/19)	15.31% (15/98)	30% (3/10)	1.003	0.789
Habitual alcohol intake, n (%)	12.80% (21/164)	8.11% (3/37)	10.53% (2/19)	9.18% (9/98)	20% (2/10)	1.792	0.608
Hypertension, n (%)	22.56 (37/164)	18.92% (7/37)	26.32% (5/19)	22.45% (22/98)	30% (3/10)	1.019	0.808
Diabetes, n (%)	13.41% (22/164)	10.81% (4/37)	15.79% (3/19)	14.29% (14/98)	10% (1/10)	0.503	0.937
Type of arrhythmia (PVCs/NSVT or VT)	136/28	30/7	16/3	83/15	7/3	1.814	0.632
VA burden on 24-h Holter ECG, beats	24,476 ± 13,429	24,423 ± 11,179	25,290 ± 13,251	25,626 ± 13,057	2574 ± 14,712	0.807	0.492
Left atrial size, cm	33.70 ± 5.22	34.75 ± 4.73	32.22 ± 6.31	33.60 ± 5.25	33.64 ± 4.38	1.021	0.385
LVEDd, cm	49.89 ± 6.17	51.92 ± 7.07	50.79 ± 7.57	48.43 ± 5.29	51.96 ± 6.80	2.635	0.068
LVEF, %	67.90 ± 7.70	67.76 ± 11.36	69.05 ± 7.14	67.28 ± 5.69	65.74 ± 7.11	0.105	0.917

Note: Abbreviation: CS, coronary sinus; GCV, great cardiac vein; DGCV_1_, the distal great cardiac vein local in epicardium of the anterior lateral wall of the mitral annulus; DGCV_2_, the distal great cardiac vein proximal to the origin of AIV. MCV, middle cardiac vein; AIV, anterior interventricular vein; Summit-CV, the communicating vein in left ventricular Summit; PLV, Posterior lateral vein; LV, lateral vein; BMI, body mass index; LVEDd, left ventricular end diastolic volume; LVEF, left ventricular ejection fraction.

**Table 2:** Procedural Outcomes.

Procedural Characteristic	Overall (n = 164)	AIV (n = 37)	Summit-CV (n = 19)	DGCV_1_, DGCV_2_, GCV, CS (n = 98)	LV, PLV, MCV (n = 10)	*χ*^2^/*F*	*p*
Procedure time, min	68.41 ± 12.89	79.26 ± 12.04 ^c^	75.45 ± 10.54 ^c^	62.03 ± 9.06 ^a,b,d^	71.54 ± 11.31 ^c^	33.558	<0.001
Fluoroscopy time, min	10.01 ± 3.79	12.51 ± 2.41 ^c^	14.40 ± 3.14 ^c^	8.03 ± 2.51 ^a,b,d^	12.29 ± 2.21 ^c^	55.958	<0.001
radiofrequency ablation lesions prior to success	3.70 ± 1.13	3.97 ± 1.56 ^c^	4.51 ± 0.96 ^c^	3.38 ± 0.84 ^a,b,d^	4.33 ± 0.84 ^c^	8.642	<0.001
duration from onset of RF energy to disappearance of IVAs, s	7.52 ± 3.43	7.72 ± 3.36	7.50 ± 3.71	7.30 ± 3.38	9.09 ± 3.71	0.871	0.457
radiofrequency ablation duration, s	144.21 ± 50.11	145.52 ± 57.59	147.02 ± 58.08	139.14 ± 50.39	145.74 ± 55.15	1.039	0.377
Activation time during VA, ms	34.91 ± 4.43	34.84 ± 4.76	34.26 ± 3.76	35.04 ± 4.67	36.37 ± 5.89	0.885	0.450
Pace mapping concordance	97.26% ± 2.83%	97.01% ± 2.88%	96.87% ± 2.34%	97.92% ± 2.43%	96.79% ± 1.82%	2.284	0.081
Distance from major coronary artery, mm	6.64 ± 1.17	5.96 ± 0.86 ^c^	6.31 ± 0.91	6.91 ± 1.11 ^a^	/	12.064	<0.001
Swartz sheath support	71.95 (118/164)	83.78% (31/37) ^c^	89.74% (17/19) ^c^	63.27% (62/98) ^a,b^	80.00% (8/10)	9.121	0.024
Accessibility of the earliest identified site	94.51% (155/164)	86.5% (32/37) ^c^	89.5% (17/19)	98.0% (96/98) ^a^	100% (10/10)	7.581	0.034
Reasons for radiofrequency ablation failure							
Proximity to coronaries (<5 mm)	4.88% (8/164)	8.11% (3/37)	5.26% (1/19)	4.08% (4/98)	/	1.250	0.647
High impedance prevents energy delivery	4.27% (7/164)	5.41% (2/37)	10.52% (2/19)	3.06% (3/98)	10.00% (1/10)	2.536	0.236
Pacing mapping activates atrium	1.22% (2/164)	/	/	2.04% (2/98)	/	NS	NS
phrenic nerve stimulation	0.61% (1/164)	/	/	/	10.00% (1/10)	NS	NS
Totally acute success	83.54% (137/164)	72.97% (27/37) ^c^	73.68% (14/19)	89.80% (88/98) ^a^	80.00% (8/10)	7.627	0.042

Note: ^a^ = compared with the AIV group, *p* < 0.05; ^b^ = compared with the summit-CV group, *p* < 0.05; ^c^ = compared with the DGCV_1_, DGCV_2_, GCV, CS group, *p* < 0.05, ^d^ = compared with the pLV, pPLV, pMCV group, *p* < 0.05.

**Table 3:** ECG characteristic of IVAS origin from the CVS.

Origin	n	The Characteristics of QRS Morphology							Transition Zone
		I	II/III/aVF	aVR/aVL	V_1_	V_2_	V_4_	V_5_~V_6_	
**Adjacent epicardial mitral annulus group**	162	R8Rs10rs61rS24qr2qs52r5	R151Rs1rs2QS8	QS152R9Qr1	R102Rs14RS20rS26	R67Rs60RS16rS19	R119Rs43	R122Rs40	
**Anterior septum subgroup**	55	Rs7r5rs7rS24qr2qs10	R55	QS55	R4Rs5RS20rS26	R9Rs11RS16rS19	R55	R55	
pAIV	36	rS24qr2qs10	R36	QS36	R1 RS13rS22	R1RS16rS19	R36	R36	V_1_7V_2_5 V_2_~V_3_15V_3_9
Summit-CV	19	Rs7r5rs7	R19	QS19	R3Rs5RS7rS4	R8Rs11	R19	R19	<V_1_6, V_1_7 V_2_~V_3_ 6
**Anterior wall subgroup**	95	Rs3rs50qs42	R95	QS95	R86Rs9	R47Rs48	R57Rs38	R61Rs34	
DGCV2	67	Rs3rs38qs26	R67	QS67	R62Rs5	R35Rs32	R57Rs10	R58Rs9	<V_1_
DGCV1	28	rs12qs16	R28	QS28	R24Rs4	R12Rs16	Rs28	R3Rs25	<V_1_
**Lateral wall subgroup**	4	rs4	R1Rs1rs2	QS2R1Qr	R4	R3Rs1	R2Rs2	R1Rs3	
GCV	2	rs2	R1Rs1	QS2	R2	R2	R2	R1Rs1	<V_1_
pLV	2	rs2	rs2	R1Qr1	R2	R1Rs1	Rs2	Rs2	<V_1_
**Posterior wall subgroup**	8	R8	QS8	R8	R8	R8	R5Rs3	R5Rs3	
pMCV	5	R5	QS5	R5	R5	R5	R4Rs1	R4Rs1	<V_1_
CS	1	R1	QS1	R1	R1	R1	R1	Rs1	<V_1_
pPLV	2	R2	QS2	R2	R2	R2	Rs2	R1Rs1	<V_1_
**distal epicardial mitral annulus group**	2	rs1qs1	R1QS1	QS1R1	QS1R1	QS1R1	R1Rs1	R1rS1	
mAIV	1	qs1	R1	QS1	QS1	QS1	R1	R1	V_2_~V_3_
mPLV	1	rs1	QS1	R1	R1	R1	Rs1	rS1	<V_1_

**Table 4:** The ECG identification of the IVAs originating from the CVS.

ECG Variables	Sensitivity	Specificity	PPV	NPV
Rs, RS or rS (with S/s wave) in lead V1 predict the VAs originating from the p-AIV and summit-CV	92.73% (51/55)	91.59% (98/107)	85.00% (51/60)	96.08% (98/102)
Rs (with S/s wave) in leads V5–V6 predict the VAs originating from the proximal regions of DGCV_1_ (including DGCV_1_, lateral wall and posterior wall subgroup)	77.50% (31/40)	92.62% (113/122)	77.50% (31/40)	92.62% (113/122)
positive wave (R, Rs or r) in lead I predict the VAs originating from summit-CV and posterior wall subgroup	74.07% (20/27)	97.78% (132/135)	86.96% (20/23)	94.96% (132/139)

PPV = positive predictive value, NPV = negative predictive value.

**Table 5:** Comparison ECG characteristics of IVAs originating from the endo-MA.

	CVS (n = 164)	Endo-MA (n = 41)	*t*	*p*
PdW, ms	61.05 ± 9.88	49.41 ± 18.38	2.510	0.021
IDT, ms	90.14 ± 19.79	65.76 ± 13.07	4.491	0.000
MDI	0.56 ± 0.09	0.47 ± 0.09	3.957	0.000
QRS duration, ms	159.57 ± 24.37	157.00 ± 26.30	0.507	0.613

PdW = the pseudo delta wave; IDT = the intrinsicoid deflection time; MDI = the maximum deflection index.

**Table 6:** The procedural complications.

Patient	Age (y)	Sex	Comorbidities	Origin	Mapping Technique	EAT (ms)	Pace Mapping	Distance from Coronary Artery, mm	RF Duration, s	RF Lesions Prior to Success, Time	Maximum Power, W	Maximum Temperature, °C	Ablation Outcome	Procedural Complication
1	56	F	coronary atherosclerosis	Summit-CV	EAT + Pace	30	11/12	5.16	13	NS	25	43	Failure	LAD stenosis
														pericardial tamponade
2	38	M	None	DGCV_2_	Pace	37	98.50%	5.1	10 + 90 × 2	3	35	43	Success	LCX spasm
3	44	F	None	AIV	EAT + Pace	37	99.20%	5.05	10 + 90	2	25	43	Success	D1 spasm
4	71	F	Hypertension	DGCV_1_	EAT + Pace	35	98.70%	7.82	5 + 60 × 3	4	30	43	Success	coronary vein rupture
														pericardial effusion
5	81	M	Hypertension	AIV	EAT + Pace	34	99.20%	8.03	10 + 60 × 3	4	25	43	Success	coronary vein rupture
														pericardial tamponade
6	62	F	diabetes	DGCV_1_	EAT + Pace	34	97.30%	6.91	15 + 90 × 2	3	30	43	Success	coronary vein dissection
7	65	M	None	DGCV_2_	EAT + Pace	33	97.20%	8.12	10 + 60	2	25	43	Success	coronary vein dissection
8	70	M	None	DGCV_2_	EAT + Pace	32	98.50%	6.19	10 + 90 × 2	3	35	43	Success	coronary vein dissection
9	69	F	None	AIV	Pace		98.90%	5.87	5 + 60 × 2	3	30	43	Success	coronary vein dissection
10	62	F	Hypertension	DGCV_2_	EAT + Pace	37	99.10%	8.76	5 + 60 × 2	3	30	43	Success	coronary vein dissection
11	54	M	None	DGCV_1_	EAT + Pace	32	100%	7.98	10 + 90 × 2	3	25	43	Success	coronary vein dissection
12	55	F	coronary atherosclerosis	DGCV_1_	EAT + Pace	35	99.50%	7.01	5 + 90 × 2	3	30	43	Success	coronary vein dissection
13	82	M	None	Summit-cv	EAT + Pace	32	95.20%	6.13	15 + 90 × 3	4	25	43	Success	coronary vein dissection
14	34	F	None	Summit-cv	EAT + Pace	45	98.40%	7.35	6 + 60 × 2	3	20	45	Success	coronary vein dissection
15	46	M	Hypertension	AIV	EAT + Pace	35	99.60%	6.15	10 + 60 × 3	4	25	43	Success	coronary vein dissection

EAT, earliest activation time; RF, radiofrequency ablation; LAD, Left anterior descending branch; LCX, Left circumflex branch; D1, the first diagonal branch.

## Data Availability

Data are available on reasonable request to the corresponding author.
